# Protein Co-Expression Analysis as a Strategy to Complement a Standard Quantitative Proteomics Approach: Case of a Glioblastoma Multiforme Study

**DOI:** 10.1371/journal.pone.0161828

**Published:** 2016-08-29

**Authors:** Evangelos I. Kanonidis, Marcia M. Roy, Ruth F. Deighton, Thierry Le Bihan

**Affiliations:** 1 SynthSys and School of Biological Sciences, Waddington building, University of Edinburgh, Edinburgh, United Kingdom, EH9 3BF; 2 Centre for Clinical Brain Sciences, University of Edinburgh, Edinburgh United Kingdom, EH16 4SB; 3 Edinburgh Medical School: Deanery of Biomedical Sciences, University of Edinburgh, Edinburgh, United Kingdom, EH8 9AG; Centre National de la Recherche Scientifique, FRANCE

## Abstract

Although correlation network studies from co-expression analysis are increasingly popular, they are rarely applied to proteomics datasets. Protein co-expression analysis provides a complementary view of underlying trends, which can be overlooked by conventional data analysis. The core of the present study is based on Weighted Gene Co-expression Network Analysis applied to a glioblastoma multiforme proteomic dataset. Using this method, we have identified three main modules which are associated with three different membrane associated groups; mitochondrial, endoplasmic reticulum, and a vesicle fraction. The three networks based on protein co-expression were assessed against a publicly available database (STRING) and show a statistically significant overlap. Each of the three main modules were de-clustered into smaller networks using different strategies based on the identification of highly connected networks, hierarchical clustering and enrichment of Gene Ontology functional terms. Most of the highly connected proteins found in the endoplasmic reticulum module were associated with redox activity while a core of the unfolded protein response was identified in addition to proteins involved in oxidative stress pathways. The proteins composing the electron transfer chain were found differently affected with proteins from mitochondrial Complex I being more down-regulated than proteins from Complex III. Finally, the two pyruvate kinases isoforms show major differences in their co-expressed protein networks suggesting roles in different cellular locations.

## Introduction

Large-scale quantitative proteomic analysis acquired under different conditions has been used to gain deeper insight into protein function and regulation [1, 2]. One widely used approach consists of comparing the level of expression of a given protein between different conditions and to determine whether or not the difference between the various groups is meaningful based on statistical analysis [3]. The following step, which consists of assigning a biological function context to the proteomics data or identifying key molecular targets, remains a challenging task. Correlation within gene expression (i.e. co-expression analysis) has been used to extract biologically meaningful information from different data sets [4, 5], but has rarely been used on proteomics data with the exception of the work of Gibbs et al 2013 [6].

Here, we have used different topologically-based strategies to divide the main list of identified proteins into different modules by first using a Weighted Gene Co-expression Network Analysis (WGCNA) developed by the Horvath group [7, 8]. These modules were, in turn, separated and broken down into clusters and sub-clusters using MCODE [9] and hierarchical clustering was applied to the protein expression patterns. As these approaches rely solely on expression profiles without *priory* functional knowledge, we then employed several knowledge-based tools to both verify and assign biological relevance to the observed sub-clusters of data. We compared the protein-protein interaction networks generated *de novo* using WGCNA against predicted networks for the same subset of proteins using STRING [10, 11] which clearly shows a significant overlap between the WGCNA analysis of the proteomics data and STRING.

In this study, we present a protein co-expression analysis of the dataset for glioblastoma multiforme previously acquired and published by Deighton *et al*. [12]. Our new findings support these previous observations. In addition to the previous findings from this study, we have highlighted three major modules of co-expressed proteins that are associated with specific membrane structures; the mitochondria, the endoplasmic reticulum (ER), and vesicle membranes. We show that within these modules, we can generate protein networks, which are similar to protein interaction networks predicted by data-mining from the literature without using an immunoprecipitation approach or native gel separation.

In addition to a major disruption of the Electron Transfer Chain (ETC) observed in the tumour samples, we show that the proteins composing each of the main ETC complexes (Complex I to IV) are mostly co-expressed but that each of the complexes are affected differently. In the ER, the unfolded protein response as well as the oxidative stress pathway are up-regulated. Furthermore, two isoforms of Pyruvate kinase (PKM) (isoform M1 and isoform M2) were differentially co-expressed with a high PKM2/PKM1 ratio supporting aerobic glycolysis (a hallmark feature of cancer) at the expense of oxidative phosphorylation (most likely inefficient due to the disruption of the ETC). While the M2 isoform seems poorly co-expressed with other proteins, the M1 isoform is part of a more defined network which is involved in ion transport, cellular response to insulin stimulus, glutamate secretion as well as syntaxin binding. In this study, we show that the use of a weighted protein co-expression analysis provides a level of information about protein interaction networks which is not possible to obtain using a standard data analysis approaches.

## Methods

The data used were the quantitative proteomics data from a glioblastoma multiforme study conducted by Deighton *et al*. [12]. All protein identities are publically available through PRIDE (http://www.ebi.ac.uk/pride) PRD000620 and the label-free quantitation output presented in S1 Table. In that study, 6 controls and 6 tumour samples were used. A mitochondrial extraction was performed, the samples were trypsinised, followed by a shotgun proteomics analysis. The quantitative analysis was performed using Progenesis (Non Linear Dynamics, UK). The MS data for this present study were searched against a human RefSeq database (34 284 sequences) using Mascot (version 2.4.1), Matrix Sciences), with a significance threshold p < 0.05 in addition to peptide ion score cut-off of 20. Each analysed protein needed at least 2 identified peptides. Conversion from RefSeq to gene symbol was performed using the biological DataBase network (bioDBnet) [13].

Label-free intensity data were ArcsinH transformed prior to analysis as log transform of 0 is not ideal. A simple trait matrix was built as follows; the parameter “state” was a single number defined as “1” for disease and “0” for control. The R WGCNA package [7] was used to perform the analysis of the data set. The Topological Overlap Matrix (TOM) was created using a cut height of 0.25 and a minimum module size of 30. The analysis produced five modules, identified with different colours (‘brown’, ‘turquoise’, ‘blue’, ‘grey’ and ‘yellow’) with the ‘grey’ module containing all proteins that were not sorted to any of the other modules shown in Table 1. A hard threshold approach was used for comparison purposes where:

*a_ij_ = corr(prot_i_, prot_j_)^β^* The correlation a_ij_ between the ArcsinH intensity of the protein prot_i_ and prot_j_ is measured. The factor β is a thresholding parameter, for hard thresholding, we used a β of 1 only for validation purposes (for FDR evaluation by comparing the same dataset against a randomised one). For the remainder of the study, we used a β of 10, justified from S1 Fig which corresponds to the lowest value showing a good scale-free topology.

**Table 1:** GO term enrichment assignments for the five main clusters.

Module	GO	GO Term	Description	P-value	FDR
	Category				q-value
**Blue**	Process	GO:0022900	electron transport chain	1.04E-26	2.98E-23
** **	Function	GO:0008137	NADH dehydrogenase (ubiquinone) activity	3.74E-15	4.85E-12
** **	Component	GO:0044455	mitochondrial membrane part	2.93E-25	2.10E-22
**Brown**	Process	GO:0006397	mRNA processing	4.28E-08	2.44E-04
** **	Function	GO:0003676	nucleic acid binding	7.16E-11	9.29E-08
** **	Component	GO:0005783	endoplasmic reticulum	1.06E-06	7.62E-04
**Turquoise**	Process	GO:0007268	synaptic transmission	4.89E-08	2.79E-04
** **	Function	GO:0030276	clathrin binding	5.95E-05	3.86E-02
** **	Component	GO:0097458	neuron part	4.80E-14	3.45E-11
**Yellow**	Process		NO ENRICHMENT FOUND		
** **	Function		NO ENRICHMENT FOUND		
** **	Component	GO:0005739	mitochondrion	4.37E-10	3.14E-07
**Grey**	Process		NO ENRICHMENT FOUND		
** **	Function		NO ENRICHMENT FOUND		
	Component		NO ENRICHMENT FOUND		

Selected groups of proteins were exported to the online Gene Ontology enRIchment anaLysis and visuaLizAtion tool (GOrilla) [14] with the gene names of each individual module used as a target set and those of the remaining modules used as a background set for the enrichment. The network data were exported to Cytoscape v3.2.1 [15], with the corresponding WGCNA function [8] where they were visualised. “Hub” clusters were defined using MCODE v1.4.1 [9]. Default suggested parameters were used.

Protein interaction networks for each module were generated from co-expression similarity using WGCNA. The same set of proteins was then clustered into a network using STRING v10 using specific confidence parameters presented in table [10, 11]. The two generated networks were then compared using “Network Analysis Tool” (NeAT) [16] with default parameters, randomisation was based on the Erdos-Renyi method. The WGCNA networks were used as the ‘Query’ networks and those from STRING as the ‘Reference’ networks. For both network types, different cut-off points were tested and are described in Table 2.

**Table 2:** Different cut-off combinations for comparing between networks generated using WGCNA and STRING prediction.

	WGCNA	STRING		
Module name^1^	Cut-off^2^	Cut-off^3^	P-value^4^	Jaccard^5^
Brown	0.3	0.4	1.40E-32	0.0871
		0.7	5.50E-20	0.081
	0.2	0.4	6.10E-63	0.1029
		0.7	4.00E-53	0.0693
	0.1	0.4	3.50E-47	0.0681
		0.7	1.40E-40	0.0377
Turquoise	0.3	0.4	1.20E-12	0.048
		0.7	2.10E-11	0.0289
	0.2	0.4	1.70E-15	0.0455
		0.7	1.60E-13	0.024
	0.1	0.4	2.30E-14	0.0427
		0.7	4.00E-11	0.0206
Blue	0.3	0.4	0.00E+00	0.2209
		0.7	0.00E+00	0.1767
	0.2	0.4	6.00E-289	0.1631
		0.7	2.40E-221	0.1137
	0.1	0.4	1.20E-154	0.1206
		0.7	5.30E-141	0.078

1) Different Modules extracted using WGCNA.

2) Threshold values for pair-wise protein co-expression (Pearson correlation^10).

3) Confidence score cut off to generate protein-protein network in STRING (http://string-db.org/).

4) p-value calculated for the overlap of the 2 protein networks (from WGCNA and from STRING) using NeAT.

5) Jaccard similarity coefficient: size of the intersection divided by the size of the union of the sample sets

**Fig 1:**
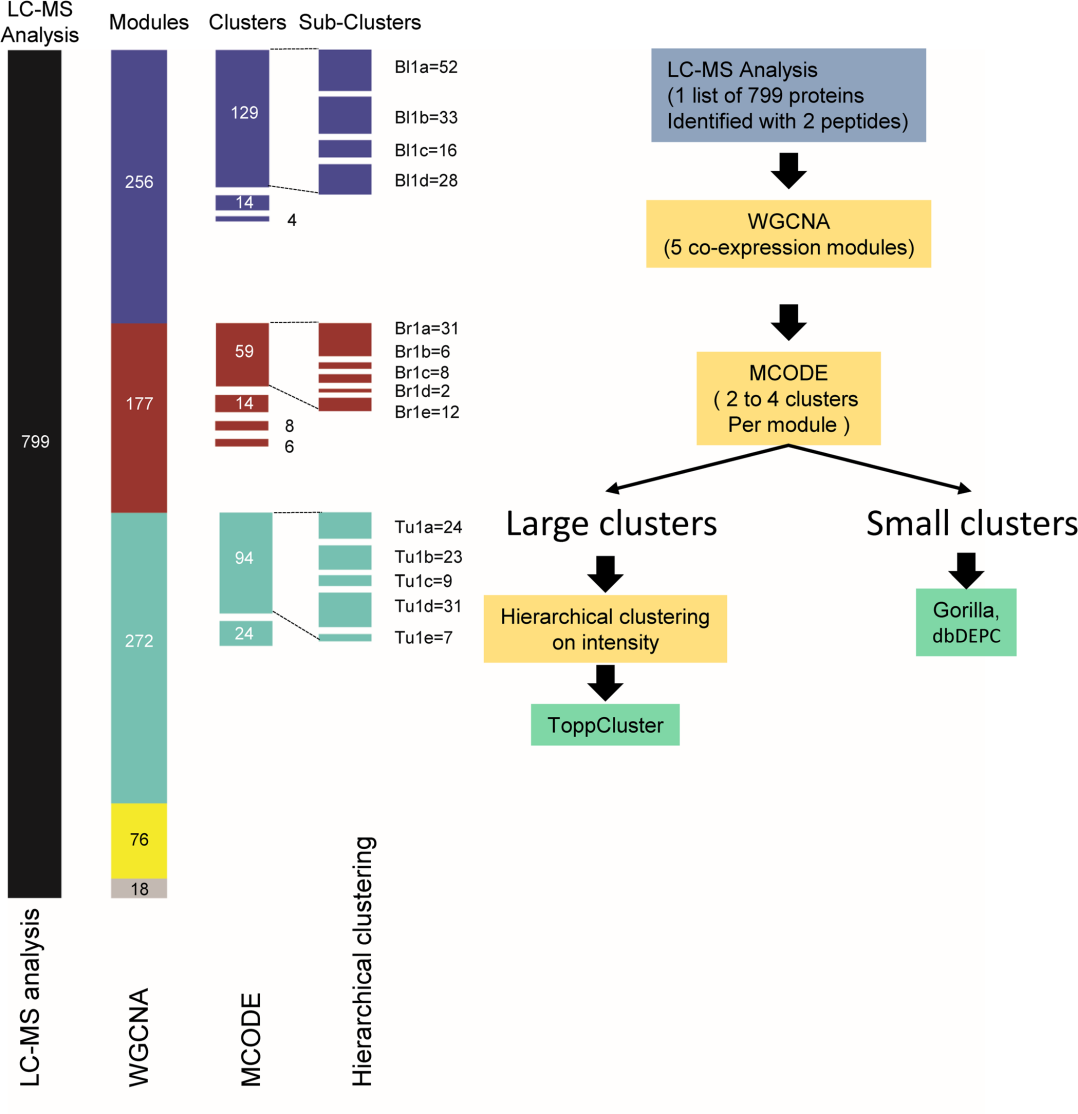
Workflow illustrating the analysis performed. On the left, the step-wise protein list fragmentation is illustrated. On the right, the different bioinformatics tools used are described. The number of proteins per group are presented and coloured using the WGCNA colour coding. The names associated to the sub-clusters are illustrated on the right side of each sub-Clusters.

Hub proteins were associated with proteins having a number of interactions which was two-fold greater than the standard deviation above the average number of interactions found in a specific module (i.e. z-score above 2). A hierarchical clustering of protein intensity was applied on the largest clusters generated from MCODE for each of the module networks using R version 3.1 GPLOT package and Ward’s method. Sub-clusters were then generated and their nature analysed using ToppCluster [17] for comparative analysis. Additionally, the clusters were analysed using the database of differentially expressed proteins in human cancers, dbDEPC 2.0 [18]. Fig 1 illustrates the overall data analysis platform used for this work and the number of proteins associated to each of the modules.

## Results

### Defining the different modules

It has been previously described that peptide and protein interaction networks possess a scale-free network topology similar to those found in gene co-expression networks [6, 19]. The glioblastoma dataset from Deighton *et al*. [12] is composed of a set of 799 proteins identified with at least two peptides (presented in S1 Table). As shown in S1 Fig, a power of β = 10 has been extracted from the original data and used for further analysis.

**Fig 2:**
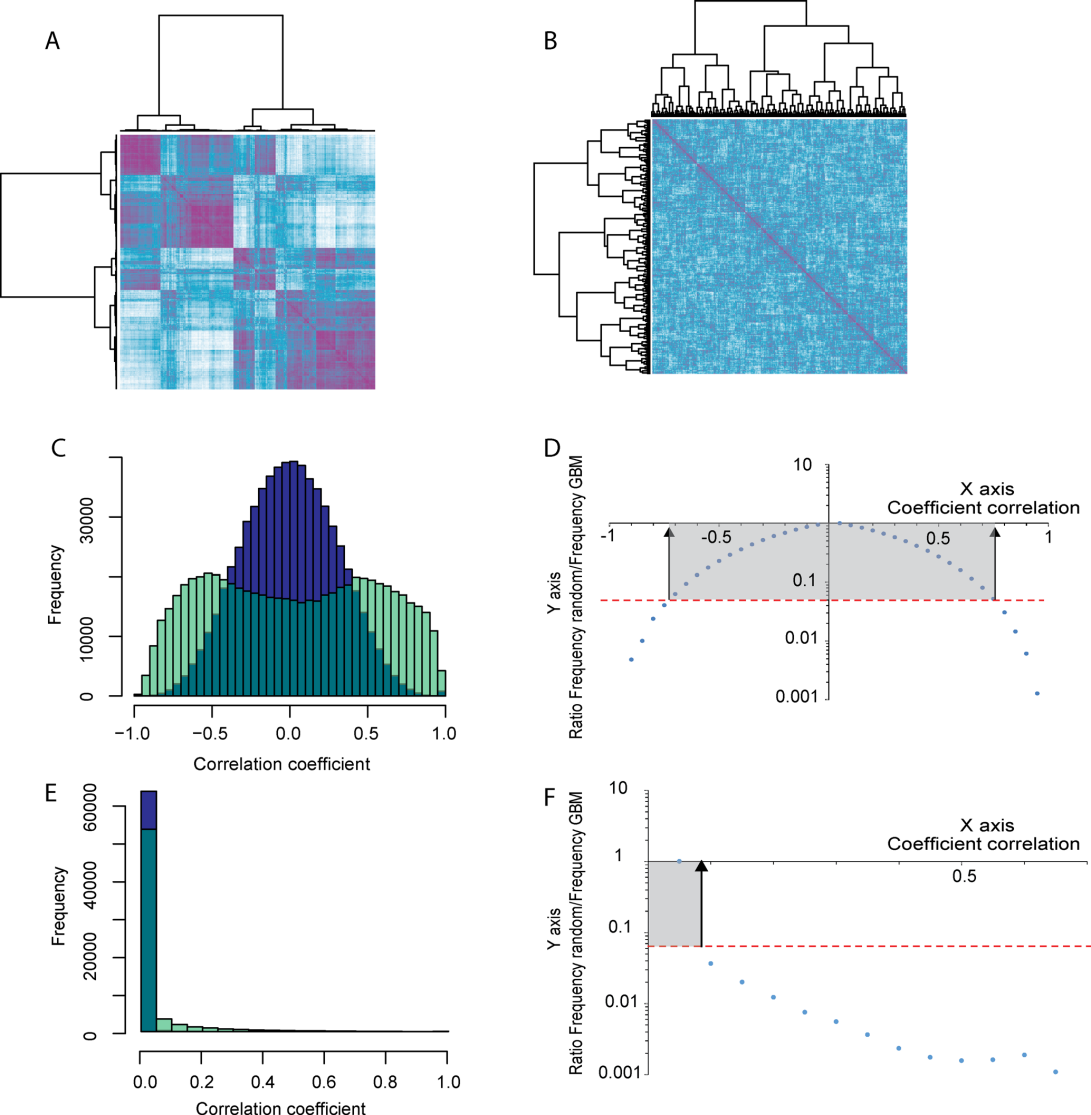
Evaluation of the confidence in the protein pair-wise measured correlation coefficient. Fig 2A hierarchical clustering of the protein pair-wise correlation coefficient for a β = 1; in Fig 2B, correlation coefficient evaluated after intensity randomisation for a β = 1. Fig 2C Distribution of the correlation coefficient from direct correlation in turquoise (extracted from Fig 2A) or after intensity randomisation in blue (extracted from Fig 2B). Fig 2D is the ratio false positive hits versus measurements obtained in the dataset. A correlation coefficient of 0.754 and above indicates positive correlation while -0.728 and less for negative correlation corresponds to a ratio of false positive below 5%. In Fig 2E the same measurements as in 2C in the case of a soft threshold β = 10. In Fig 2F, a false positive rate equal to or below 5% corresponds to a value of 0.0993 and above.

The overall data analysis strategy used in this work is presented in Fig 1. A series of data analysis tools, based on either network topology characteristics or literature knowledge was used to cluster groups of proteins. One possible concern with the use of the Deighton et al (10) dataset for network analysis is its rather small size (a 2 group comparison with 6 replicates only) where normally datasets of at least 25–30 samples are commonly used for co-expression analysis (4). We have estimated the false discovery rate (FDR) by using a permutation approach as described elsewhere [20, 21]. Hierarchical clustering of the pair wise correlation coefficient was evaluated first using a thresholding parameter of β = 1, shown in Fig 2A each row and column are represents proteins, the colour purple is associated with clusters having a high correlation coefficient, and white is associated to a high anti-correlation coefficient. Protein intensities were also randomly permutated and the same clustering method was used once again (shown in Fig 2B). As expected, a significant decrease in the level of correlation is observed. The plot of the distribution of the correlation coefficients for both datasets (the direct dataset and the randomised one) is shown in Fig 2C. The randomised dataset is centred around 0 (blue) while the normal dataset (turquoise) exhibits two distributions roughly centred on 0.5 and -0.5 associate to either correlated or anti-correlated pairs or proteins, respectively. We have evaluated the FDR for different correlation coefficients and a FDR of 5% was calculated for a correlation coefficient of 0.754 and above and values of -0.728 and less for meaningful anti-correlation (Fig 2D). A similar calculation was performed using a β = 10 (Fig 2E), a FDR of 5% was found for value an a_ij_ of 0.0993 and above (Fig 2F).

**Fig 3:**
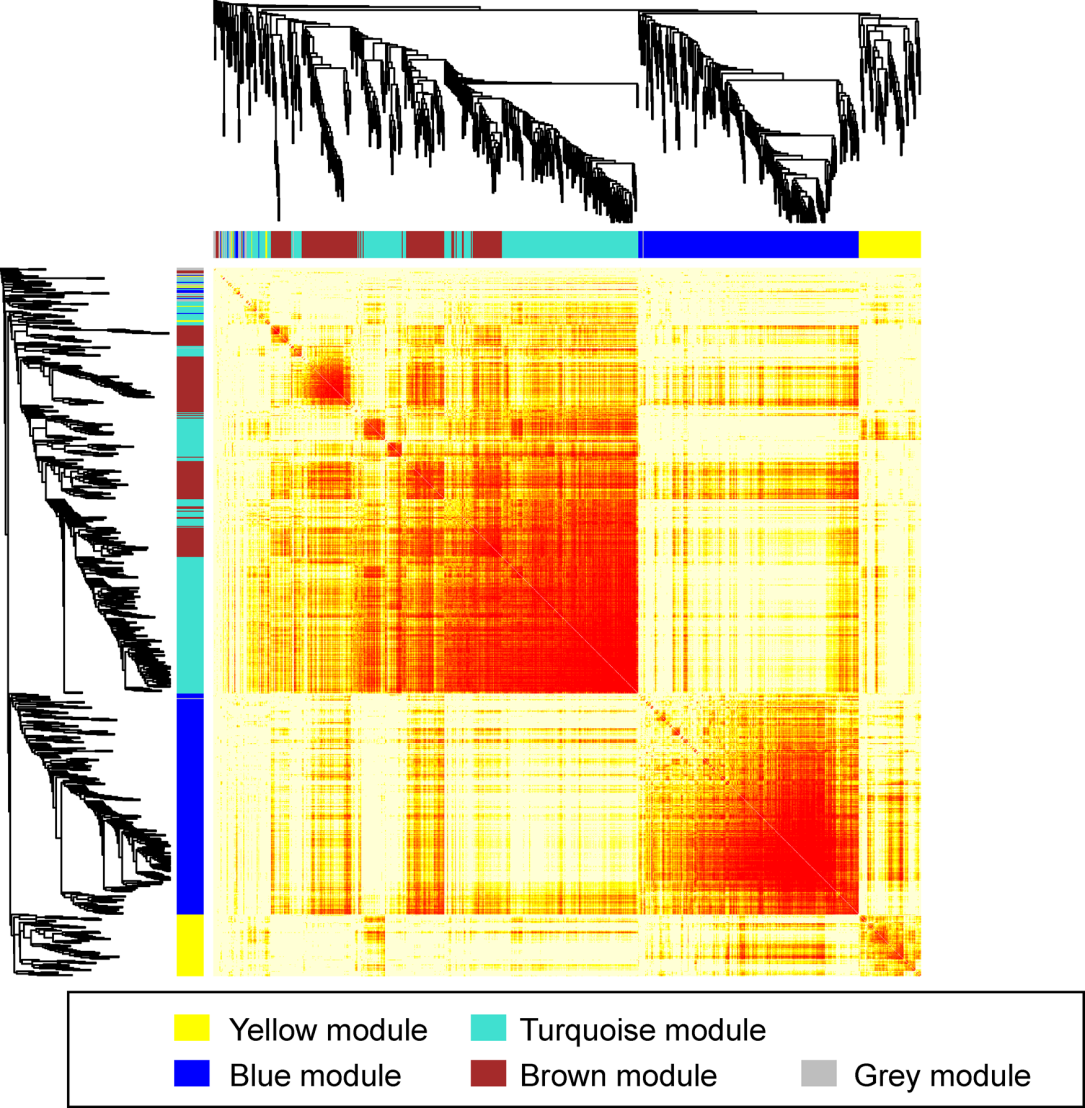
Clustering of the proteomic label-free analysis of glioblastoma multiforme. The data shows five major clusters. The clustering heatmap was created using a soft thresholding of β = 10 on the entire proteomics dataset. Data clustering and module membership generation are described in Materials and Methods. The scale ranges from yellow to red, with yellow demonstrating low topological overlap and red representing high topological overlap.

The initial analysis was based on the WGCNA package for R [7, 8]. Fig 3 shows the Topological Overlap Matrix (TOM) plot applied to the dataset from Deighton *et al*. [12]. Each row and column represents proteins. The colours red and yellow indicate the high and low weighted correlation values, respectively, and are assigned by the TOM-based dissimilarity between each protein co-expression level. Each of the five modules, represented by the coloured bar on the top and left of the matrix (blue, yellow, brown, turquoise, and grey), are associated to set of proteins sharing a high value for co-expression level. Only 18 proteins did not cluster into a module and were allocated to the grey module. The turquoise module is the largest one, containing 272 proteins, followed by the blue module with 256 proteins, the brown module with 177 proteins, and the yellow module with 76 proteins (illustrated in Figs 1 and 3).

Similar to gene expression patterns, proteins within a given module are co-expressed with higher correlation than with proteins from different modules. We then asked if those proteins which are part of the same cluster share some similarities in terms of biological function. To address this, we performed a Gene Ontology (GO) enrichment analysis for each module. The proteins from each cluster were analysed using Gorilla.

Table 1 shows the results of the GO enrichment analysis. Both, the yellow and the grey modules do not show any major functional enrichment. On the other hand, the three other modules clearly show significant GO terminology enrichment. They represent primarily three different components, the blue module being associated with the mitochondrial membrane part (q-value of 2.10e-22), the brown module being associated with the ER (q-value of 7.62e-4) and the turquoise module being associated with the neuronal part/membrane vesicles (q-value of 3.45e-11), suggesting that the proteins from a given cellular location have similar function and display a higher degree of co-expression.

**Fig 4:**
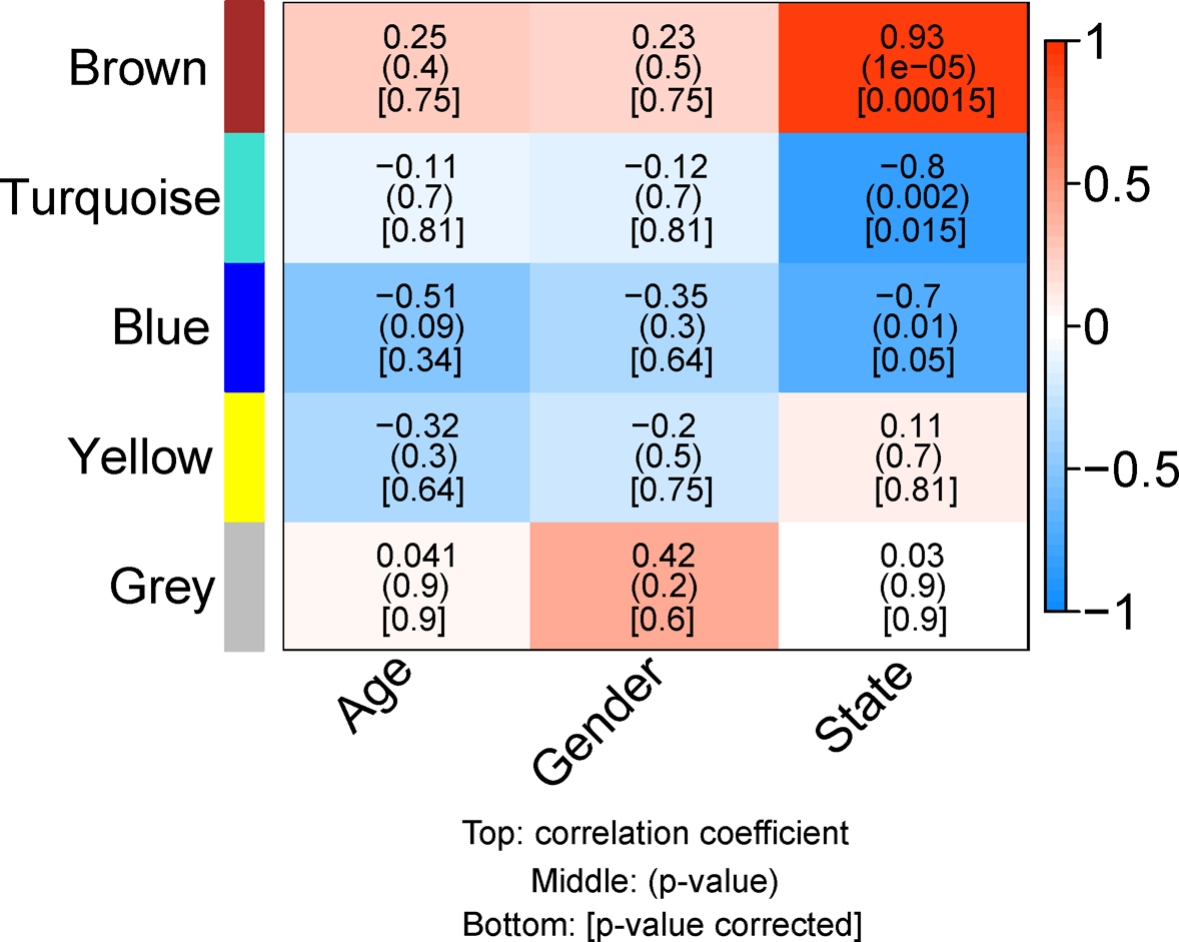
Data-trait correlation between the first principal component (Eigengene) of each module (y-axis) and the clinical traits (x-axis). All positive correlations are shown in red and the negative correlations are shown in blue. The correlation coefficients between cells are shown and p-values are displayed within brackets below the correlation coefficient itself. The modules with the lowest and highest significant p-values are the brown, the turquoise, and the blue module.

The five main protein modules were then correlated to phenotype data (trait matrix) to highlight possible trends. This step was performed in order to identify possible links between the clusters of proteins and higher-level information. The phenotype information is presented in S2 Table. Fig 4 is a heatmap showing the correlation between the five modules and three different traits. The traits used for correlation were general traits (age and gender of patient taken from Deighton *et al*. [12] and state (extracted from S2 Table). The numbers within the heatmap squares show Pearson correlation coefficients quantifying the correlation between the modules and the phenotype traits. The numbers in brackets are the respective p-values and corrected p-values, respectively.

In the two modules having the lowest functional information (yellow and grey), no significant correlations were found. No significant correlation was found with Age and Gender for any of the modules. The brown module (ER) is anti-correlated to both the blue (mitochondrial membrane) and turquoise modules (membrane vesicles). The brown module shows a high level of correlation with the state (i.e. control = 0, tumour = 1) (r = 0.93). These results indicate that the proteins within the brown module are mostly up-regulated in glioblastoma tumour samples. The turquoise module shows strong anti-correlation with state whereas the blue module shows a similar, but less pronounced anti-correlation with the state.

### Validating the protein networks generated from WGCNA

High values of co-expression between two proteins may be predictive of protein- protein interactions. In order to assess the validity of the interactions generated with the presented analysis, the networks that were generated using WGCNA were compared with networks of known interactions obtained from STRING for the same protein dataset. The statistical comparison between the two different approaches was performed using the Network Analysis Tool NeAT (see Materials and Methods section for detailed description). Table 2 shows the results of the comparison between WGCNA and STRING outputs for different threshold values. The Jaccard coefficient was used to determine the similarity between two sample sets.

A combination of different parameter thresholds for both the WGCNA analysis and STRING was tested in order to optimise the best overlap of the two independent approaches to predict protein-protein interactions (illustrated in Table 2). The parameter threshold for WGCNA is the minimal threshold Pearson’s correlation coefficient of co-expressed paired proteins. The STRING score is defined as the confidence in the interaction between two protein nodes. Different combinations of parameters have been used and their effect on network overlap (Jaccard coefficient) and prediction quality (shown by p-value) is shown in Table 2. In addition, we have evaluated the similarity between the STRING output and a randomised pairing (using the same node but having randomised the same number of edges predicted by WGCNA for a given cut-off). The chosen combination of cut-off was based on several factors including minimal p-value and Jaccard score as shown in Table 2 and the highest difference in p-value obtained between the WGCNA and a randomised similar data set against STRING.

The best threshold combination appears to be 0.3 (associated to a FDR of 0.5% and less) for WGCNA and 0.4 for the confidence score generated by STRING (expressed as 0.3/0.4 pair in the text), which gives the higher Jaccard value for the blue and turquoise modules. On the other hand, for the brown module the optimal threshold combination seems to be 0.2 for WGCNA(which is associated to a FDR of 1% and less) and 0.4 for the confidence score in STRING (0.2/0.4 pair). However, more pronounced differences between WGCNA prediction and a random dataset were observed with a cut-off of 0.2/0.4 for the brown and turquoise modules. We have calculated a p-value of 6e-289 for the blue module against STRING, whereas a randomised dataset under the same condition had a p-value of 1.2e-13. In the brown module, we measured a p-value of 6.1e-63, whilst a random dataset generated a p-value of 9.1e-9. In the turquoise module we observed 1.2e-12 whilst a randomised dataset generated a p-value of 3.3e-7. Although low p-values were observed with randomised datasets using STRING, they were largely different from what was predicted with the real dataset. Those highly significant p-values for the random datasets are a consequence of a high ratio of number of edges *versus* nodes. The higher this ratio, the less of an effect the edge position randomisation has on the predicted network.

**Fig 5:**
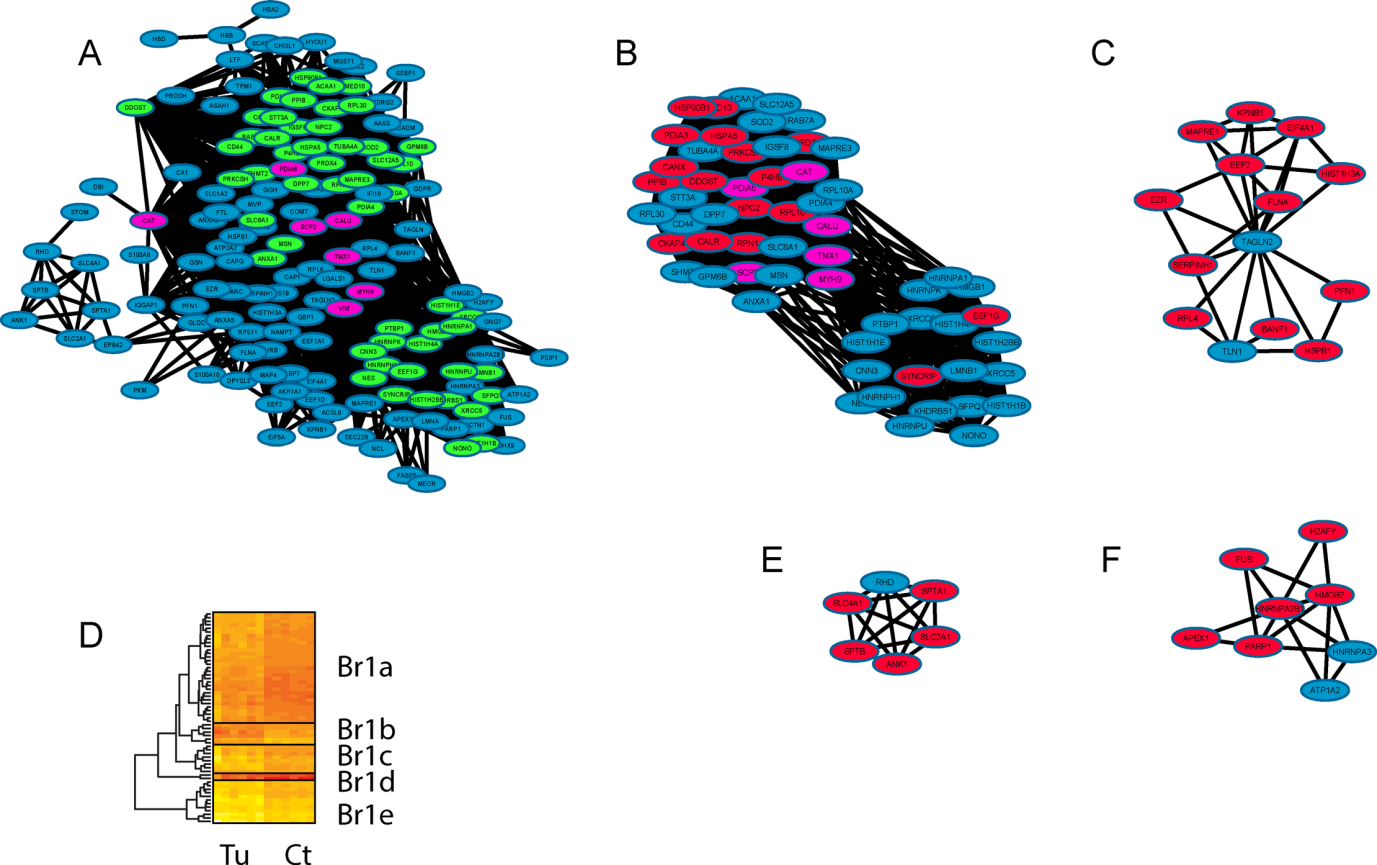
Visualisation of the brown module using a network generated in Cytoscape. The global network is shown in Fig 5A. The main large cluster identified by the MCODE application, being coloured in green and the most interconnected (‘hub’) proteins shown in purple. This main cluster extracted from the brown module is shown in Fig 5B and other secondary clusters identified by MCODE are also shown (Fig 5C, 5E and 5F). In Fig 5B, 5C, 5E and 5F, proteins highlighted in red are associated with a defined GO term assigned by GOrilla. The main cluster as shown in Fig 5B was analysed using a hierarchical clustering approach based on protein intensity across the tumour (Tu) and the control (Ct) samples and is shown in Fig 5D. Five sub-clusters were identified and further analysed using Toppcluster.

**Fig 6:**
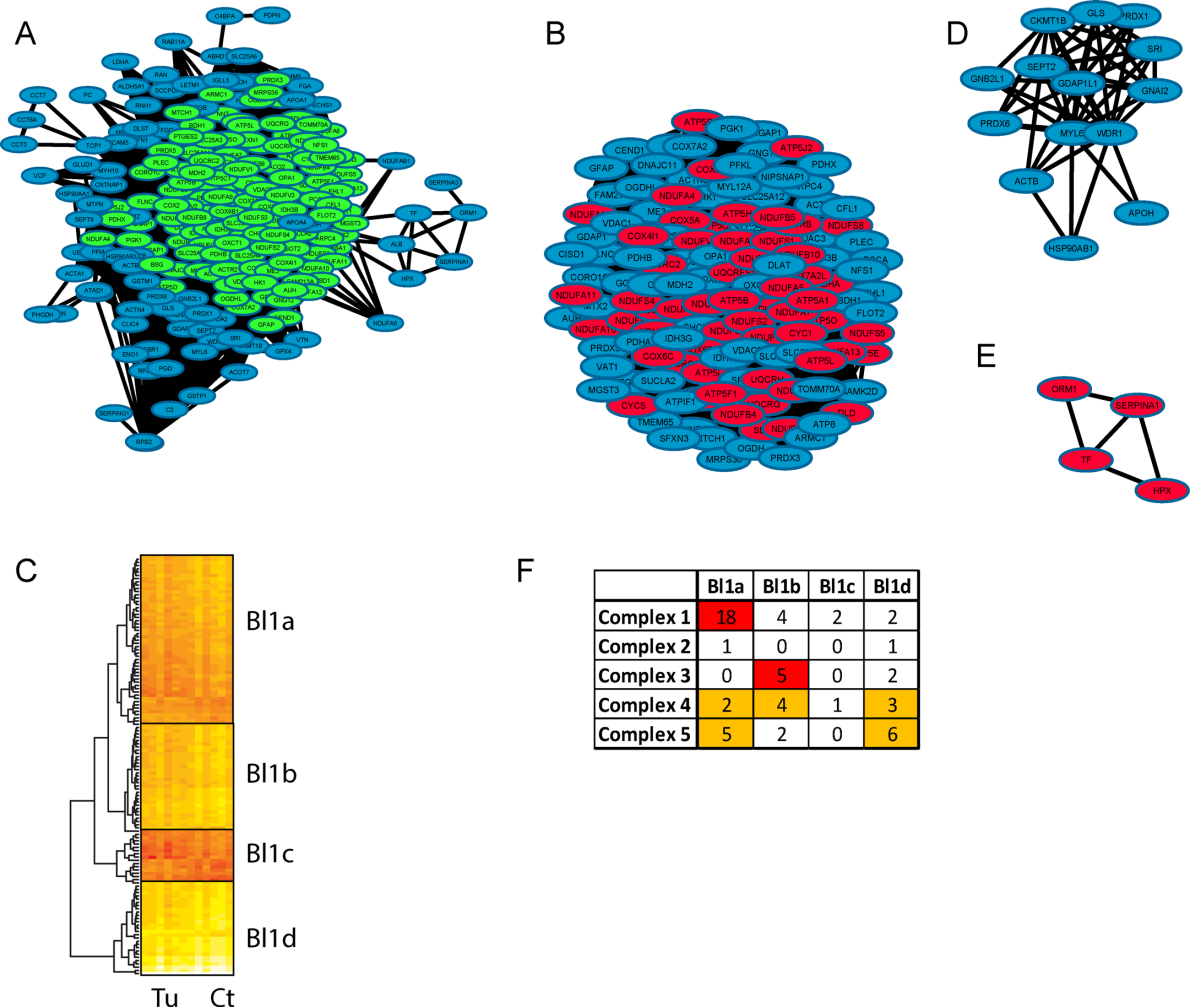
Visualisation of the blue module using a network generated in Cytoscape. The global network is shown in Fig 6A with the main large cluster, identified by the MCODE application, being coloured in green. The main cluster extracted from this module is shown in Fig 6B and other secondary clusters identified by MCODE are also shown in Fig 6D and 6E. In Fig 6B, 6D and 6E, proteins highlighted in red are parts of a defined GO term according to GOrilla. The main cluster as shown in Fig 6B was analysed using a hierarchical clustering approach based on protein intensity across the tumour (Tu) and the control (Ct) samples and is shown in Fig 6C. Four sub-clusters were identified. Distribution of the proteins from the five complexes across the different sub-clusters is shown in Fig 6F.

**Fig 7:**
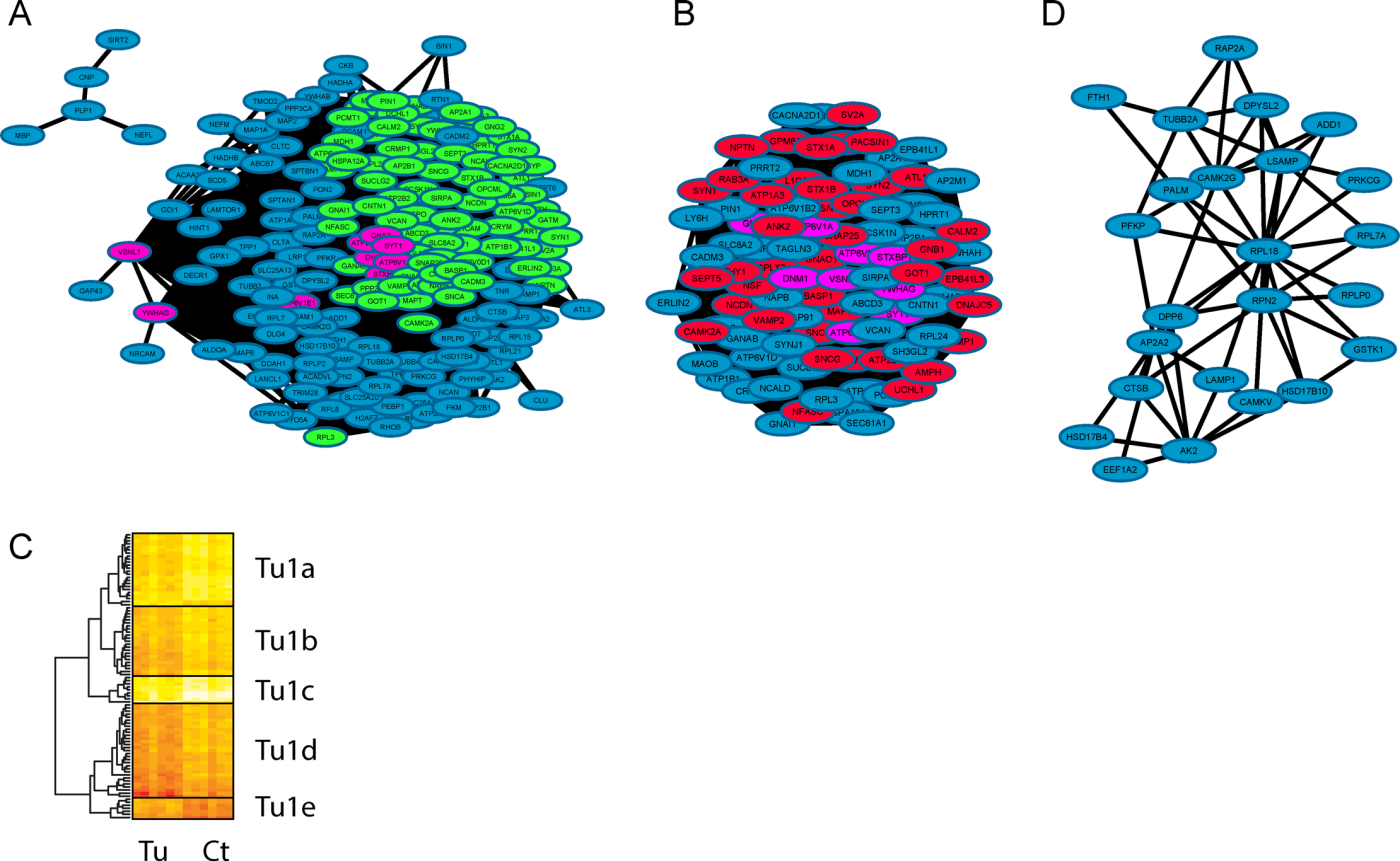
Visualisation of the turquoise module using a network generated in Cytoscape. The global network is shown in Fig 7A which contains a large (Fig 7B) and a small (Fig 7C) network. In Fig 7A, the main large cluster, identified by the MCODE application, is coloured in green and the most interconnected (‘hub’) proteins are visualized in purple. The main cluster extracted from this module is shown in Fig 7B and a smaller secondary cluster identified by MCODE is shown in Fig 7D. In Fig 7B, proteins highlighted in red are part of a described biological function according to GOrilla, no specific enrichment has been found for cluster 7D. The main cluster as shown in Fig 7B was analysed using a hierarchical clustering approach based on protein intensity across the tumour (Tu) and the control (Ct) samples and is shown in Fig 7C.

In general, the number of interactions predicted by WGCNA was significantly higher compared to what has been reported in STRING. The resulting outcomes are densely interconnected protein networks. In order to reduce the dimensions of the three main large modules identified and extract more subtle information regarding their nature, we used other topological based tools. MCODE [9], a tool that identifies highly interconnected nodes within a complex network, was used to isolate smaller groups of proteins (which will be referred to as a “cluster”) within each of the three main modules (blue, brown and turquoise module) and identify key highly connected proteins (i.e. Hubs). For each module, a major dense cluster was identified and several minor clusters were also generated (Figs 5, 6 and 7).

### Partitioning the modules; cluster and sub-clusteranalysis

Figs 5–7 show network representations of each module. Figs 5A, 6A and 7A are the global networks for the brown, blue and turquoise modules, respectively. Coloured in purple are the highly connected hub proteins for each module (the blue module has no identified protein hub). In the global network, proteins that belong to the first cluster generated by MCODE (as presented in Figs 5B, 6B and 7B, respectively) are coloured in green in Figs 5A, 6A and 7A.

The three main clusters in Figs 5B, 6B and 7B were still densely interconnected, with an overlap of 59 out of 177 proteins for the brown module (33%), 129 out of 256 proteins for the blue module (50%) and 94 out of 272 proteins for the turquoise module (35%). As those main clusters represent an important part of each module, they are mostly an enriched version in terms of function and protein localisation of each module. In addition to those main clusters, several smaller clusters were as well identified and are described below.

Some smaller clusters for the three modules (see Figs 5C, 5E, 5F, 6D and 6E) showed significant GO term enrichment based on GOrilla (node coloured in red). The main terms describing the most significant enrichment varied in most cases, but were mainly found to be well described by cellular component and biological process GO terms.

In order to identify subtle variation within each main cluster, we applied hierarchical clustering on the protein intensity for each of the main clusters (i.e. the large clusters in Figs 5B, 6B and 7B), which highlighted some possible sub-clustering. These heatmaps are shown in Fig 5D for the brown module, Fig 6C for the blue module and Fig 7C for the turquoise module. Each of those clusters and sub-clusters were analysed using the comparative tool Toppcluster.

#### Description of the ER (Brown) Clusters and Sub-clusters

From the initial 177 proteins composing this module, 154 proteins had at least one WGCNA co-expression parameter above 0.2 the threshold value we used for that clusters/sub-clusters. From those 154 proteins, 87 proteins are sub-grouped into four clusters. The 67 proteins that did not associate with any cluster were also not assigned any major biological function. A group of seven proteins were identified as “hub” proteins, i.e. proteins which are highly interconnected (shown in purple in Fig 5A and 5B). Those proteins are CAT, PDIA6, CALU, SCP2, TMX1, MYH9, and VIM, of which PDIA6 and TMX1 are involved in disulphide isomerase activity and SCP2, CAT and VIM in peroxisome signalling.

In Fig 5B, the proteins highlighted in red are associated with cell compartment GO terminology “ER”. In Fig 5E, the term used to describe the proteins in red was the GO Cellular Component “cell cortex part” (ANK1, SLC2A1, SLC4A1, SPTA1, SPTB) primarily involved with cytoskeletal protein binding, while the subgroup ANK1, SPTA1, SPTB is also related to biological processes associated with the tetrapyrrole and porphyrin-containing compound biosynthetic process. RHD is the only protein not associated with the cell cortex part, but is linked to the plasma membrane along with the other proteins in this cluster.

For Fig 5C, the “cell cortex” and “cortical cytoskeleton” are over-represented in the cellular compartment GO terminology (EZR, FLNA, MAPRE1), the proteins EZR, FLNA, PFN1, TLN1 are involved in maintenance of protein location while the large group of proteins containing EEF2, EIF4A1, EZR, FLNA, HSPB1, KPNB1, MAPRE1, PFN1, RPL4, SERPINH1 share the molecular function “poly(A) RNA binding”. The term used to describe the proteins in red was the GO Function term “nucleic acid binding”. One can notice that two proteins, were not characterised by the prevalent GO term (i.e., protein TAGLN2 and TLN1 both in blue in Fig 5C). These two proteins are associated with actin binding. However, TAGLN2 is a poorly characterised protein without a determined function.

Fig 5F shows proteins involved in poly(A) RNA binding (APEX1, FUS, HMGB2, HNRNPA2B1, HNRNPA3, PARP1), and most of the proteins found in this cluster are primarily located in the nucleoplasm (Cellular compartment). They are: APEX1, FUS, H2AFY, HMGB2, HNRNPA2B1, HNRNPA3 and PARP1. The term used to describe the proteins in red was the GO Function “DNA binding.”

The main brown cluster illustrated in Fig 5B contains proteins enriched in the ER part, with proteins involved in ER stress. Some specific domain enrichments were found, such as Thioredoxin-like fold (EEF1G, P4HB, PDIA3, PDIA4, PDIA6, PRDX4, TMX1) and ER targets (CALR, HSP90B1, HSPA5, P4HB, PDIA4, PDIA6, PRKCSH). The three main pathways represented in these data are:

1. mRNA processing (HNRNPA1, HNRNPH1, HNRNPK, HNRNPU, NONO, PTBP1, SFPQ, TMED10)
2. Protein processing in ER CALR, CANX, CKAP4, DDOST, HSP90B1, HSPA5, P4HB, PDIA3, PDIA4, PDIA6, PRKCSH, RPN1, STT3A
3. Calnexin/calreticulin cycle (CALR, CANX, PDIA3, PRKCSH)

The main cluster shown in Fig 5B was separated into five sub-clusters, Br1a to Br1e, of 31,6,8,2 and 12 proteins, respectively (shown in Fig 5D). Mainly the two sub-clusters Br1a and Br1e generate functional information. The ER parts are found in the Br1a and Br1e sub-cluster with CALU, CKAP4, DDOST, PDIA4, PRKCSH, RPN1, STT3A, TMED10, TMX1 for Br1a.

Proteins associated with mRNA processing were found in the sub-cluster Br1a (HNRNPA1, HNRNPH1, HNRNPK, HNRNPU, NONO, PTBP1, SFPQ) and nucleoplasm (HNRNPA1, HNRNPH1, HNRNPK, HNRNPU, LMNB1, NONO, PTBP1, SFPQ, XRCC5, XRCC6). Interestingly, the pair XRCC5 and XRCC6 were identified, which play a major role in the non-homologous end joining (NHEJ) pathway [22].

Proteins associated to the cytoplasmic membrane-bound vesicles are unique to the Br1e sub-cluster (CALR, CANX, HSP90B1, HSPA5, P4HB, PDIA3 and PPIB). In addition, unique proteins associated to calcium ion binding such as ANXA1, CALR, CANX, HSP90B1, HSPA5 are found in the sub-cluster Br1e which also contains unique proteins involved in response to ER (CALR, HSP90B1, HSPA5, P4HB, PDIA3). Proteins found in this last subgroup (CALR, HSP90B1 and especially HSPA5) are well known to be involved in the activation of signalling protein activity and unfolded protein response (UPR).

#### Description of the Mitochondrial (Blue) Clusters and Sub-clusters

From the initial 256 proteins composing this module, 214 proteins have a WGCNA co-expression parameter above 0.2. From those 214 proteins, a group of 147 proteins can be sub-divided into three clusters. The 67 proteins that are not part of any major cluster are not significantly co-expressed, however, they did share some biological function such as fibrinogen Complex FGA, FGB, FGG, FN1 and are parts of the Integrin signalling linked to the MAP kinase pathway by recruiting Grb2 to the FADK1/SRC activation complex. In contrast to the majority of protein in this module, this small subset of proteins is up-regulated in the tumour samples.

Fig 6A shows the main module Blue while Fig 6B is associated to the main blue cluster generated by MCODE. The main cluster in Fig 6B is largely composed of proteins involved in the ‘Electron Transport Chain’. The small cluster (Fig 6E) (HPX, ORM1, SERPINA1, TF) is associated to the cellular component “extracellular space”. The larger network (Fig 6D) has no significant functional enrichment according to GOrilla, although STRING significantly associates (p-value of 2.059e-5) all of its proteins to the extracellular region, except for GLS, CKMT1B and GDAP1L1. This module also contains three proteins having a thioredoxin fold domain (GDAP1L1, PRDX1, and PRDX6). The cluster in Fig 6D contains several proteins which have been associated with a variety of different cancer types including breast cancer which are WDR1, PRDX1, PRDX6 [23], and HSP90AB1 [24], hepatocellular carcinoma HSP90AB1, PRDX1, PRDX6 [25], gastric cancer WDR1, HSPB90AB1[26], cervical cancer PRDX1, HSP90AB1 [27], thyroid cancer HSP90AB1, PRDX6 [28], prostate cancer HSP90AB [29], and colorectal cancer WDR1 [30].

The main blue cluster from Fig 6B can be separated into four sub-clusters (shown in Fig 6C), Bl1a to Bl1d, consisting of 52, 33, 16, and 28 proteins, respectively. The smallest cluster (Bl1c) had little biological information deduced. The blue cluster 1 (Fig 6B) is mainly composed of proteins associated to the mitochondrial respiratory chain which, in turn, comprises Complexes I to V. These different complexes are co-expressed slightly differently and are therefore distributed across the four sub-clusters (Fig 6F). The Complex I proteins are mainly found in sub-clusters Bl1a (18 proteins out of 26 identified in this study), proteins from Complex III are mainly found in Bl1b (5 out of 7 proteins identified in this study), Complex IV is found across Bl1b and Bl1d while Complex V is distributed between sub-clusters Bl1a and Bl1d. Only two proteins from Complex II were identified (SDHA and SDHB) that were not found to be part of the same sub-cluster.

#### Description of the Neuronal (Turquoise) Clusters and Sub-clusters

From the initial 272 proteins composing this module, 198 proteins have a WGCNA co-expression parameter above 0.3. From those 198 proteins, a group of 118 proteins is involved in two clusters. The 80 proteins not part of any major clusters although not significantly co-expressed shared some biological function such as fatty acid beta oxidation (ACAA2, ACADS, ACADVL, DECR1, ECI2, HADHA, HADHB), gluconeogenesis (ALDOA, ENO2, PGAM1, SLC25A1, SLC25A13), and glucose metabolism (ALDOA, ENO2, PGAM1, PKM1, SLC25A1, SLC25A13).

The main turquoise module (Fig 7A) generated both a large and a small network while using a threshold of 0.3 for the WGCNA coefficient. The large main network (Fig 7A) is composed of proteins involved in different “membrane vesicles” structures whilst the small network is mostly related to the myelin sheath (CNP, MBP, PLP1, SIRT2). The overall module containing the “neuronal part” is associated with proteins assigned the terms endocytic vesicles and cytoplasmic membrane-bounded vesicles with some ATPase and GTPase activity; furthermore, a subgroup of proteins is associated to glial cell differentiation (CNP, GAP43, MBP, PLP1, TPPP).

A group of proteins which are highly connected (i.e. Hub proteins; VSNL1, YWHAG, ATP6V1E1, ATP6V0A1, GNAZ, SYT1, DNM1, ATP6V1A, STXBP1) were identified. Three ATPase H+ transporting lysosomal units were found to be quite interconnected and are involved in several different functions (e.g. ATP hydrolysis coupled proton transport and ferric ion transport). Three proteins combined with SYT1, DNM1 and STXBP1 are part of the synaptic vesicle cycle.

In Fig 7C, the main cluster in 7B has been divided into five sub-clusters of 24, 23, 9, 31, and 7 proteins, respectively, (Tu1a to Tu1e). According to Toppcluster, mainly three sub-clusters show biological enrichments which are Tu1a, Tu1b and Tu1d. The sub-cluster Tu1a is rich in proteins involved in ion/cation transport (ANK2, ATP1B1, ATP6V0A1, ATP6V1A, ATP6V1B2, CAMK2A, CNTN1, NSF, SNAP25, STX1A, STX1B, SYT1, THY1, and YWHAZ). In addition, proteins from cluster Tu1a have molecular functions associated to SNARE binding (NSF, SNAP25, STX1A, STX1B, and SYT1).

A group of three proteins from Tu1a is involved in regulation of mitochondrial membrane permeability (CAMK2A, YWHAG, and YWHAZ). The sub-cluster Tu1b is rich in proteins involved in pathways associated to coated vesicle membrane and clathrin-coated vesicle (AP2A1, AP2M1, DNAJC5, SNAP91, and VAMP2), while Tu1d is mainly composed of proteins involved in synaptic vesicle endocytosis and synaptic vesicle recycling (AMPH, RAB3A, SH3GL2, SNCA, SYNJ1, and SYP).

The data presented in Fig 7D showed no strong functional enrichment after being analysed by GOrilla, although according to Toppcluster and STRING the following proteins are associated to protein targeting to ER as biological process: RPL18, RPL7A, RPLP0, and RPN2. Additionally, STRING identified several proteins as parts of membrane-bound vesicle from this cluster (CAMKV, CAMK2G, PALM, RPLP0, RAP2A, RPL7A, GSTK1, TUBB2A, AP2A2, DPYSL2, FTH1, PFKP, DPP6, and AK2).

#### Pyruvate kinase isoforms co-expression network

**Fig 8:**
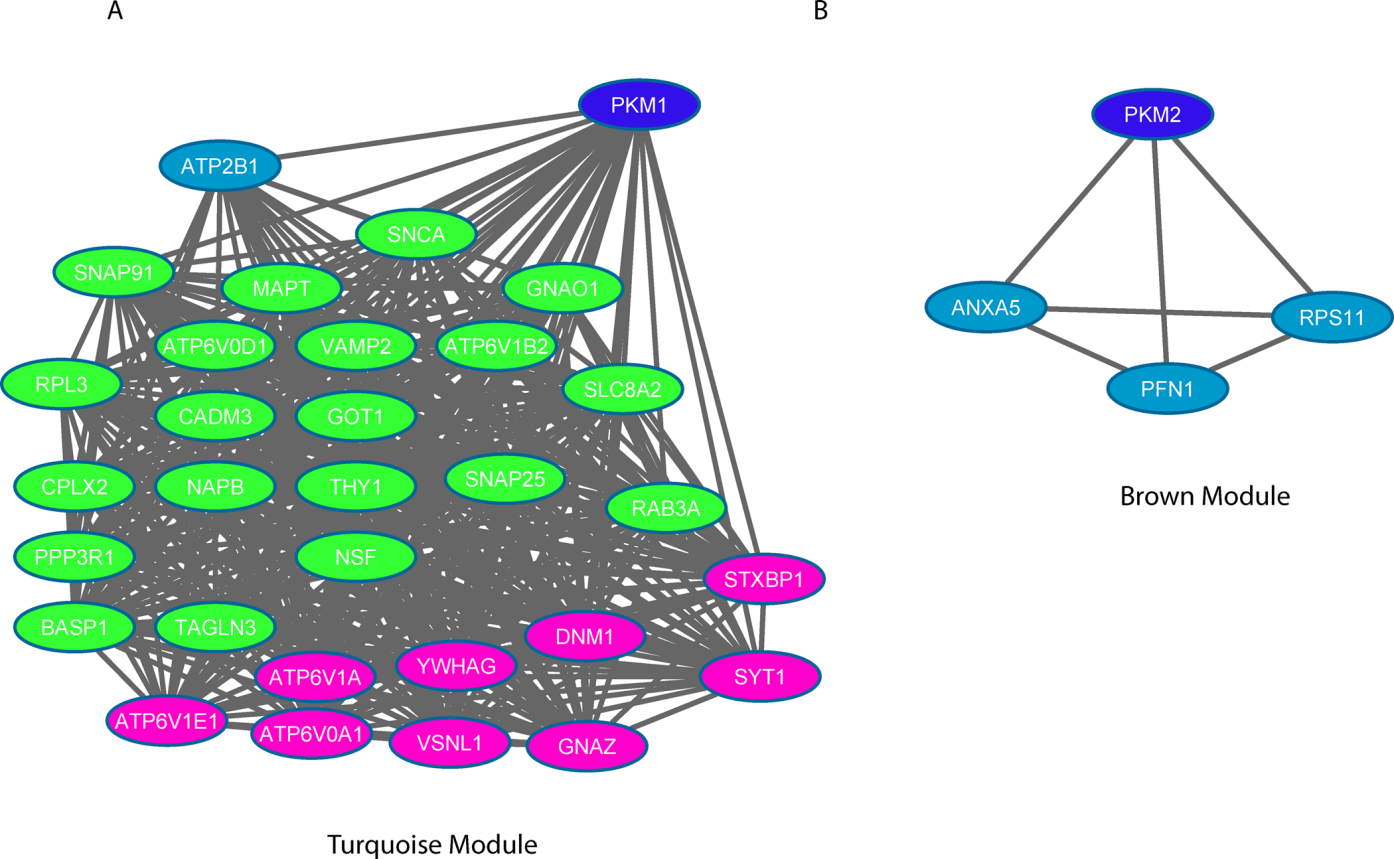
**Pyruvate kinase isoform M1 (Left) and M2 (Right) and their respective co-expression networks (direct interactors only).** Nodes in dark blue are the 2 PKM proteins, in pink are proteins defined as HUB proteins from the turquoise Module. The proteins in green are proteins associated to the larger sub-cluster presented in Fig 7.

The two isoforms of PKM (PKM1 and PKM2) were identified (Fig 8). While PKM1 was found to be down-regulated and associated to the turquoise module, the PKM2 isoform was up-regulated and associated to the brown module. The direct co-expressed proteins for each pyruvate kinase protein isoform are illustrated in Fig 8. Twenty-nine proteins were found to be co-expressed with PKM1 while only three showed co-expression with PKM2 in this study.

Regarding PKM1, the largest group of proteins exhibiting direct co-expression are those related to the synaptic vesicle cycle (KEGG Pathway): ATP6V0A1, ATP6V0D1, ATP6V1A, ATP6V1B2, ATP6V1E1, CPLX2, DNM1, NSF, RAB3A, SNAP25, STXBP1, SYT1, and VAMP2.

Several other metabolite-associated groups of proteins were identified, such as proteins related to cellular response to insulin stimulus (YWHAG, VAMP2, ATP6V0A1, ATP6V0D1, ATP6V1A, ATP6V1B2, ATP6V1E1, and GOT1), glutamate secretion (VAMP2, SYT1, SNAP25, STXBP1, and RAB3A), and to syntaxin binding (CPLX2 NAPB NSF SNAP25 STXBP1, and VAMP2).Some of the highly correlated expression profile proteins with PKM1 include guanine nucleotide binding protein (GNAO1) and syntaxin binding protein 1 (STXBP1) for which no known direct interaction has been reported yet. In a similar manner, the protein cell adhesion molecule 3, CADM3 involve in the calcium-independent cell-cell adhesion molecules is as well highly correlated with PKM1 but no known relation between CADM3 and PKM1 has been previously reported. Both cases merit to be explored by studying the role of both PKM in specific tissues, in this cases, PKM1 role in the synaptic vesicle. For the PKM2 cluster, no significant term enrichment was found.

## Discussion

In the present study, we used a combination of different analytical methods to characterise protein co-expression measured from a quantitative proteomics analysis. The main method (WGCNA) was applied to the Deighton et al dataset [12] and allowed for the subgrouping of all proteins into five main modules, of these modules, three are associated with membrane-based organelles. The soft threshold power used in this study (β = 10) is in the same order of magnitude as that used in previous work [31]. This approach has resulted in identification and sub-grouping of proteins by their distinct features into three different cellular locations.

Although the dataset is of a modest size (a total of 12 experiments), we have shown that it was possible to extract valid and meaningful information. We evaluated the FDR for different correlation coefficient thresholds using the same dataset but with the position of each intensity for a given protein being randomised. The threshold values selected to generate the different networks in Figs 5–7 have a FDR of between 0.5% and 1% which is quite conservative. The 3 major modules clearly show significant enrichments thus supporting the validity of the approach even on small datasets. The networks generated using WGCNA have been compared to the knowledge-based method STRING and shows the overlap between the two independent methods to be significant. Thus, we have shown that the use of WGCNA to generate protein networks *de novo* without the need for an immunoprecipitation-based approach. These networks could not have been generated with the initial type of analysis used in Deighton et al [12] The over-represented GO term to describe functional enrichment was mainly the cellular component with the blue module’s proteins being significantly localised in the mitochondria. The brown module was enriched in ER proteins and the turquoise module enriched in various types of vesicle membranes. In addition, these abundance of the proteins in these modules correlated to traits which included the relative increase, or decrease of expression in cancer tissue (Fig 4). The brown module (ER) proteins correlate with proteins that are up-regulated in tumour samples, while the blue module (mitochondrial membrane part) and turquoise module (membrane vesicles) both correlate with proteins that are down- regulated in tumour samples.

The original proteomic analysis reported by Deighton *et al*. [12] was based on a mitochondrial fraction enrichment. However, in this current study we have identified several proteins from other membrane-based organelles such as the ER and vesicular membranes. Although these membrane fractions share similar physical properties to the mitochondrial fractions and could introduce complexity to the samples, their identified interaction networks reveal the broader of the many effects of glioblastoma multiforme. In addition, the concomitant enrichment of ER in the mitochondrial fraction might be a result of those two organelles being interconnected through mitochondria-associated membranes (MAM) [32] a finding that may provide a deeper understanding of intra-cellular organelle coordination during tumorigenesis.

Despite the use of a soft threshold β = 10 to generate the different networks, these networks were significantly denser than what was predicted by STRING. Although the overlap between STRING and WGCNA was found to range between 5 and 22%, the calculated p-values clearly support that the observed networks were not simply due to chance (p-values between 1.2e-12 to 6e-289).

One observation, also reported in Deighton *et al*. [12] is that the electron transfer chain (ETC) is significantly down-regulated in cancer cells (part of the blue module). Proteins from the major complexes of the ETC were identified in this study and were found to be mostly down-regulated. This observation was supported by electron microscopy showing that the inner membrane of the mitochondria is severely disrupted [12]. However, in this manuscript we have found that the different complexes were marginally co-expressed in different sub-clusters especially for Complex I (70% of Complex I proteins were found in sub-cluster Bl1a) and Complex III (70% of Complex III proteins found in sub-cluster Bl1b) which suggests that these two complexes are not affected in the same way, with Complex I proteins being slightly more down-regulated than the proteins from Complex III. A similar observation on the different effects on complexes of the ETC has been made on mitochondrial fractions isolated from a transgenic mouse model [33]. A disruption of the electron transfer chain and oxidative phosphorylation could potentially lead to elevated ROS generation [34]. Several proteins involved in the oxidative damage response were also found to be up-regulated such as catalase, superoxide dismutase 2, peroxiredoxin 1, 4 and 6.

Several key proteins involved in the “ER stress response” or the “unfolded protein response” (UPR) were found to be up-regulated. The disruption of the ETC and the up-regulation of several proteins involved in oxidative stress support a link with cellular events such as protein oxidation and protein folding. Oxidative stress and ROS generation are important components of the ER stress response. The major enzymatic components of ROS production during UPR induction are protein disulfide isomerase (PDIA4 was found up-regulated in this study); ER proteins involved in stress response were found significantly co-expressed (CALR, HSP90B1, HSPA5, P4HB, and PDIA3) specifically in the sub-cluster Br1e. Most of these proteins were also found up-regulated during oxygen and glucose deprivation for 18h [35] which supports an integrated cellular survival response. Furthermore, mitochondrial HSP90 has been reported to play an important role in controlling core metabolic processes by stabilising Complex II of the ETC and allowing cellular respiration to continue under compromised conditions, contributing to tumorigenesis [36].

Cells under normal conditions have a basal level of ROS, which is intrinsic to signalling mechanisms. However, an increase of ROS levels is observed upon exposure to specific stress such as cytotoxic reagents, irradiation, and environmental pollutants and during some specific enzymatic reactions such as: mitochondrial respiratory chain reactions, activity of glucose oxidase, amino acid oxidase, xanthine oxidase, and NADP/NADPH oxidase). Triggering of the unfolded protein response (UPR) consequential to the exposure to oxidative stress is most likely a mechanism to preserve both cell function and survival. On the other hand, continuous oxidative stress and protein misfolding induce apoptotic pathways and play crucial roles in the pathogenesis of multiple human diseases including diabetes, atherosclerosis, and neurodegenerative diseases.

HSPA5 (also known as GRP78, Bip) is a chaperone protein whose expression is significantly enhanced under various conditions including glucose deprivation, oxidative stress, treatment with Ca2+ ionophores, and hypoxia [37]. Higher levels of HSPA5 are essential for sustaining cell viability under specific kinds of stress. The up-regulation of stress proteins in tumour cells has been shown to inhibit programmed cell death and to contribute to drug resistance [37]. Therefore, HSPA5 has some potential as a novel therapeutic target for both anti-tumor and anti-angiogenesis activity [38].

Similar to the blue module, the turquoise module is mainly composed of proteins which are down-regulated under tumour-forming conditions and are mainly enriched in “vesicle membrane” fractions. The main cluster in Fig 7B contains most of the proteins having known biological functions. Surprisingly, the YWHAZ protein was found to be down-regulated in our study, whilst Nishimura et al. [39] observed that YWHAZ-overexpression plays a major role in tumour cell proliferation. One of the highly interconnected protein members of the hub proteins was YWHAG, which is a 14-3-3 adapter protein involved in the regulation of a broad spectrum of signalling pathways. YWHAG binds to a large number of partners, usually by recognition of a phosphoserine or phosphothreonine motif. Binding generally results in the modulation of the activity of the binding partner by protein kinase C inhibitor activity. A protein kinase C (PRKG) was also found co-expressed in the turquoise module. Both, 14-3-3 protein YWHAG and YWHAZ in combination with CAMK2A were found in the same sub-cluster Tu1a and are involved in the regulation of mitochondrial membrane permeability.

The soft threshold method used in this study (β = 10) significantly reduced the importance of module interconnection. However, a few interesting proteins were identified in the ER which are more strongly co-expressed with proteins in the mitochondria including PDIA6 and HSPA5/GRP78 which are known to play a crucial role on apoptosis inhibition [38, 40]. Regarding the blue module, a few proteins were found to be highly co-expressed with other proteins outside the module suggesting a co-ordination role far beyond their immediate environment. One of the identified proteins is CKMT1B, which was also found to be down-regulated in squamous cell carcinomas and in clinical samples [41].

A component of the turquoise module is the isoform 1 of pyruvate kinase (PKM1), which was highly co-expressed with more proteins than its counterpart PKM2 from the brown module (Fig 8). It has often been described in the literature that the PKM protein expression switches from PKM1 to the PKM2 isoform during tumourigenesis [42, 43]. We observed a change in isoform ratio where the PKM1 isoform is down-regulated with a ratio tumour/control = 0.26 associated to the turquoise module. While the PKM2 isoform is up-regulated (ratio tumour/control = 2.14 and clustered in the brown module). The observed changes in this current study, although meaningful, do not support a complete shift from one isoform to the other one as described by Bluemlein *et al*.[44]. The two isoforms of PKM are differentially expressed (M1 and M2) with the different co-expression network proteins of each isoform supporting an increase in aerobic glycoysis at the expense of oxidative phosphorylation (rendered inefficient due to the disruption of the ETC).

Decreasing the PKM2/PKM1 ratio has recently been described as a therapeutic strategy in patients with glioblastoma multiforme [45]. As shown in Fig 8, co-expression of PKM2 was limited to only three other proteins (ANXA5, PFN1, and RPS11). Conversely, PKM1 was found co-expressed with more than 30 other proteins from the turquoise module which are mostly involved in ion transport, cellular response to insulin stimulus, glutamate secretion as well as syntaxin binding; a common theme among these proteins is related to the synaptic vesicle cycle with 12 out of the 32 proteins being directly involved in this pathway. Although a broad range of functions is associated to the different proteins co-expressed with PKM1, our findings support that pyruvate kinases are possibly bound to synaptic vesicles with substrates that may be supporting vesicular glutamate uptake [46]. In addition, several of the highly PKM1 co-expressed proteins reported in this study were newly identified. Guanine nucleotide binding protein (GNAO1) and syntaxin binding protein 1 (STXBP1) and the protein cell adhesion molecule 3, CADM3 involved in the calcium-independent cell-cell adhesion molecules has not been previously reported and merit to be explored by more tissue targeted analysis of both PKM.

It is intriguing that the 2 PKM isoforms show expression patterns which are not co-localised; PKM2 found mostly co-expressed with proteins from the mitochondrial fraction while PKM1 found co-expressed with proteins related with vesicular membrane. In summary, protein co-expression analysis of the mitochondrial protein fraction revealed novel protein networks with several intrinsically linked functions and uncovered functional modulesHere we have shown and validated with several different strategies that a weighted protein co-expression analysis complements more conventional approaches based on differentially expressed proteins from different groups and can serve as a valuable method for revealing new trends and information clustering which are impossible to capture otherwise.

## Supporting Information

S1 Fig Soft threshold parameters and resulting scale-free topology. (DOC)

S1 Table Complete proteomics dataset used in this study (proteins identified with at least 2+ peptides). (XLS)

S2 Table Table of the trait matrix used for the correlation with the modules. (XLS)
