# Supplementary material for: Protein Co-Expression Analysis as a Strategy to Complement a Standard Quantitative Proteomics Approach: Case of a Glioblastoma Multiforme Study
Source: PLoS One. 2016 Aug 29;11(8):e0161828. doi: 10.1371/journal.pone.0161828 (PMC5003355; doi:10.1371/journal.pone.0161828)
Supplement: S1 Fig — (DOC) [file pone.0161828.s001.doc]

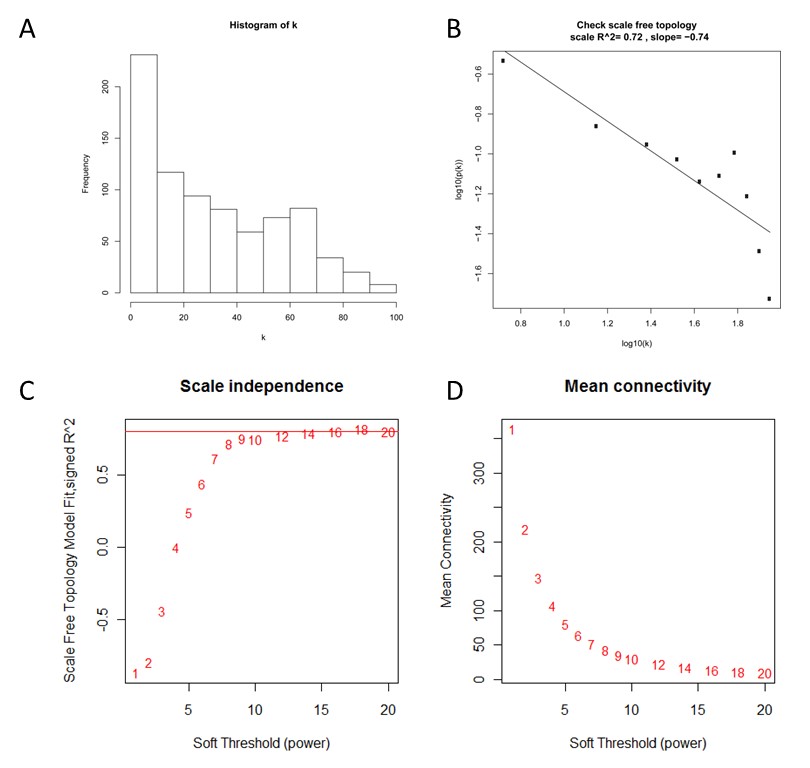


**S1 Fig.** Network parametrisation. **S1A Fig** segment left quartile, the histogram of the network connectivity resulting from the choice of a threshold power (β=10). Log-log plot of the complete-network connectivity distribution in **S1B Fig**. The x-axis shows the logarithm of whole network connectivity, y-axis the logarithm of the corresponding frequency distribution. The trend follows a straight line, which is referred to as a scale-free topology. Soft threshold power value satisfying the scale-free topology (**S1C Fig**) and associated connectivity (**S1D Fig**). The red line in **S1C Fig** shows the outcome using a h=0.8. The numbers are associated to the numerical power (soft threshold) value used.
